# Subboiling Moist Heat Favors the Selection of Enteric Pathogen* Clostridium difficile* PCR Ribotype 078 Spores in Food

**DOI:** 10.1155/2016/1462405

**Published:** 2016-06-07

**Authors:** Alexander Rodriguez-Palacios, Sanja Ilic, Jeffrey T. LeJeune

**Affiliations:** ^1^Division of Gastroenterology and Liver Disease, Digestive Health Research Institute, Department of Medicine, Case Western Reserve University School of Medicine, Cleveland, OH 44106, USA; ^2^Department of Human Sciences, Human Nutrition, College of Education and Human Ecology, The Ohio State University, Columbus, OH 43210, USA; ^3^Food Animal Health Research Program, Ohio Agricultural Research and Development Center, The Ohio State University, Wooster, OH 44691, USA

## Abstract

Emerging enteric pathogens could have not only more antibiotic resistance or virulence traits; they could also have increased resistance to heat. We quantified the effects of minimum recommended cooking and higher temperatures, individually on a collection of* C. difficile* isolates and on the survival probability of a mixture of emerging* C. difficile* strains. While minimum recommended cooking time/temperature combinations (63–71°C) allowed concurrently tested strains to survive, higher subboiling temperatures reproducibly favored the selection of newly emerging* C. difficile* PCR ribotype 078. Survival ratios for “ribotypes 078” :  “other ribotypes” (*n* = 49 : 45 isolates) from the mid-2000s increased from 1 : 1 and 0.7 : 1 at 85°C (for 5 and 10 minutes, resp.) to 2.3 : 1 and 3 : 1 with heating at 96°C (for 5 and 10 minutes, resp.) indicating an interaction effect between the heating temperature and survival of* C. difficile* genotypes. In multistrain heating experiments, with PCR ribotypes 027 and 078 from 2004 and reference type strain ATCC 9689 banked in the 1970s, multinomial logistic regression (*P* < 0.01) revealed PCR ribotype 078 was the most resistant to increasing lethal heat treatments. Thermal processes (during cooking or disinfection) may contribute to the selection of emergent specific virulent strains of* C. difficile*. Despite growing understanding of the role of cooking on human evolution, little is known about the role of cooking temperatures on the selection and evolution of enteric pathogens, especially spore-forming bacteria.

## 1. Introduction

One of the most important spore-forming human pathogens of the last three decades is enteric bacterium* Clostridium difficile* [1]. In the 1980–90s it was considered a pathogen exclusively associated with hospitals [2], but infection severity and incidence have escalated also affecting healthier communities [3, 4]. Based on PCR ribotyping there are hundreds of* C. difficile* strains. Of concern, spores of common pathogenic strains for humans (PCR ribotypes 027, 078, 017, 001, 077, 014, and 033) have been found in the food supply since 2004 [5, 6]. Although there are still no verifiable reports of foodborne* C. difficile* transmission, identifying multidrug-resistant PCR ribotype 027 strains in foods in 2004 was of concern because of their emergence as hypervirulent in Europe and North America [7–9]. Initially unrecognized, PCR ribotype 078 has recently received much attention [6, 10–15]. Widely found in food animals and the food supply, this new hypervirulent* C. difficile* has tripled its incidence among affected people in recent years [6, 10–15].

Using validated protocols we previously determined that, in order to achieve 6.5 log_10_ reductions of* C. difficile* spore counts (to meet food safety standards, based on* Salmonella*), it is necessary to heat foods at temperatures higher than 71°C (160°F), which is the minimum food safety temperature typically recommended for cooking most foods [16, 17]. Using validated protocols, we also showed that heating at 63 and 71°C (recommended minimum temperatures for seafood and hamburgers, resp.) increased* C. difficile* recovery by promoting germination (awakening) of up to 30% more spores [16]. Heating at 96°C (205°F, subboiling) inhibited 99.9% of spores within a couple of minutes (95% confidence interval of *D*-value_96_ = 0.8, 1.4); however, some spores remained unaffected after extended heating or recovered growth after being thermally inhibited [16].

Given the high prevalence of* C. difficile* in retail foods (from seafood to poultry; 2–42%) [11, 18, 19] with notable predominance of PCR ribotypes 027 (up to 27%) and 078 (up to 73–100%) in North America [6, 11, 15, 18–20], as well as the variable resistance of* C. difficile* to heat inactivation [16, 21, 22], we hypothesized that thermal stress during cooking could destroy some* C. difficile* strains favoring the systematic selection of others. Here we tested that hypothesis by moist heating: first a collection of* C. difficile* isolates, and then three distinct strains (individually and as a mixture of strains heated in the same experimentally inoculated food-ground beef-item) to statistically estimate the comparative survival probabilities of selected genotypes at and above recommended cooking temperatures, specifically at 63 and 71°C; 85 and 96°C.

## 2. Materials and Methods

Three experiments were conducted with spores of* C. difficile* prepared and aged on phosphate buffered saline for 52 weeks as previously described and validated for clostridia [16, 24]. First, we individually tested 94 animal-derived isolates of* C. difficile* from studies in the mid-2000s [25–28]. Briefly, suspensions of 10^5^ spores (~100,000) in 110 *μ*L of phosphate buffered saline (PBS) were individually heated at 85 and 95°C for 0, 5, and 10 minutes using 96-well PCR plates and a thermocycler as described [16]. These time-temperatures represent an extended spectrum of options above the minimum food safety cooking recommendations available in North America (Table 1).

Next, we used three prototypic strains to individually determine if the thermal inhibition (curves) was similar when heated at 85°C (185°F; minimum recommended temperature for “difficult-to-cook” meats, Table 1). The strains selected correspond to the first PCR ribotypes 078/toxinotype-V and 027/toxinotype-III isolated from cattle in 2004 [14], and a type strain from the American Type Culture Collection isolate ATCC-9689/toxinotype-0 associated with* C. difficile* infections (CDI) in humans, which was deposited in the ATCC bank in the 1970s [29]. Freshly frozen (3% fat content) retail ground beef was used as heating matrix. Briefly, duplicate random 6-gram beef samples mixed with approximately 7 log_10_ *C. difficile* spores per 0.1 grams in 600 *μ*L PBS suspensions (*i.e.*, to moisten the meat and reduce air pockets for better heat transfer at a ratio of 100 *μ*L of suspension: 1 g of beef). Inoculated and controls beef aliquots (with only PBS; noninoculated) were then placed, flattened manually, and rolled-sealed after removing air pockets inside Whirlpack bags, which were heated and kept submerged at the bottom of automated water baths (15 cm depth with space on all sides to allow even heating) simultaneously preset at respective temperatures as previously described [16, 17]. To synchronize the immersion and removal of the beef-containing Whirlpack bags, each water bath and sets of samples were closely monitored by a designated researcher. Timing started when duplicated chilled beef aliquots fitted with automatic thermal sensors indicated that the center of the beef inside the Whirlpack bags reached the target temperature in each water bath (the higher 96°C temperature bath required longer preheating time, compared to 63°C and 85°C). A set of nonheated aliquots were left at 4°C and used for control baseline purposes (time 0). After 10, 20, and 30 minutes of heating, the bags were removed and immediately submerged in a container full of chilled icy water (4°C) where the bags remained until enumeration, which was conducted for the entire batch less than 14 hours later. Survivor spores were enumerated using 10-fold PBS serial dilutions and plating on tryptic soy 5% defibrinated sheep blood agar after 48–72 hours of incubation in an anaerobic incubator at 37°C with an atmosphere composed of 10% carbon dioxide, 10% hydrogen, and 80% nitrogen (Whitley Workstation DG250, Microbiology International, Inc.). The experiment was duplicated with strain 078 to verify reproducibility and internal validity. Log transformed spore counts were analyzed using generalized linear regression models (outcome: log data; independent variables: strain, time points, temperature, and replica).

Lastly, we tested the prototypic strains from the previous experiment expanding the study using a randomized complete block design to quantify the relative and comparative survival probability for each individual strain as they were heated together in the same heat matrix (beef) as a 1 : 1 : 1 ratio strain mixture, at concentrations comparable to the previous experiment (6.9 ± 0.6 log_10_ *C. difficile* spores per 0.1 g of beef heated in water baths inside Whirlpack bags). Spore growth inhibition was tested as a function of time-temperature and fat content of the beef used as heating matrix. In short, we assessed 63, 71, 85, and 96°C and used 3 and 30% fat content retail ground beef (which was plated in 2-fold PBS serial dilutions to determine the concentration of naturally present background* C. difficile*, if any) and assessed the effect of heat on naturally present (non-*C. difficile*) anaerobic accompanying microbial flora (also referred to as retail background beef microbiota). The beef was refrigerated until the day of experimental spore inoculation and heat testing, one day prior to the product expiry date, which would contain the maximum permissible concentration of retail background microbiota to be used as internal control for comparison purpose for heat inhibition of non-spore forming vegetative bacteria. Spore inoculation and heat testing of* C. difficile* were conducted as in the previous experiment. In short, inoculated beef and noninoculated control beef aliquots were heated and enumerated after 0, 2.5, 5, 7.5, 10, 15, 20, and 30 minutes to determine the number of* C. difficile* colony-forming units over time as an indirect measure of spore survival. To determine relative survival probabilities, up to 25 survivor* C. difficile* colonies were selected from representative serial dilutions and generated a genetic fingerprint for each colony selected by extracting the DNA from purified single colonies and then conducting single-colony PCR ribotyping as previously described [30]. PCR ribotyping was the method of choice for genotyping because this method clearly distinguishes strains of distinct origin based on the unique gel electrophoretic fingerprints for the strains selected in this experiment. Isolation frequency data were analyzed using generalized linear regression as described above. Survival (yes/no) data controlling for beef aliquot were analyzed to predict adjusted survival probabilities using multivariable multinomial logistic regression in which the outcomes for logistic probabilities were the three strain categories (PCR 078, 027 and the ATCC-9689 isolate), with the remaining parameters as variables [31].

Comparisons of heat resistance across pathogens are largely based on strains tested in isolation with *D*-values derived for single temperatures and limited integration of data over a range of temperatures. *D*-values correspond to the units of time needed to inhibit 90% (one log_10_) of a microbial population at a given temperature. Derived from linear equations that describe the slope of the (straightest) exponential part of the inhibition curves, *D*-values ignore the curve shoulders. The *Z*-value, a less intuitive number, corresponds to the temperature units needed to move one unit in a log transformed *D*-value linear scale plotted against corresponding *D*-value temperatures. Although the *Z*-value attempts to integrate time-temperature dynamics, the approach is purely linear and void of the ability to illustrate nonlinear interactions that could exist between heating protocols and the strain type. Based on extended heating experiments [21], here, we focused on the extreme right shoulder of the inhibition curves which represent the most heat-resistant colony-forming spores and performed multinomial regression analytics which are unusual in food safety studies. In this context, survival (yes/no) data was analyzed using logistic regression. Exact odds ratios were estimated for actual and hypothetical sample sizes. Stata software was used (v10.1, College Station, TX).

## 3. Results

**Figure 1:**
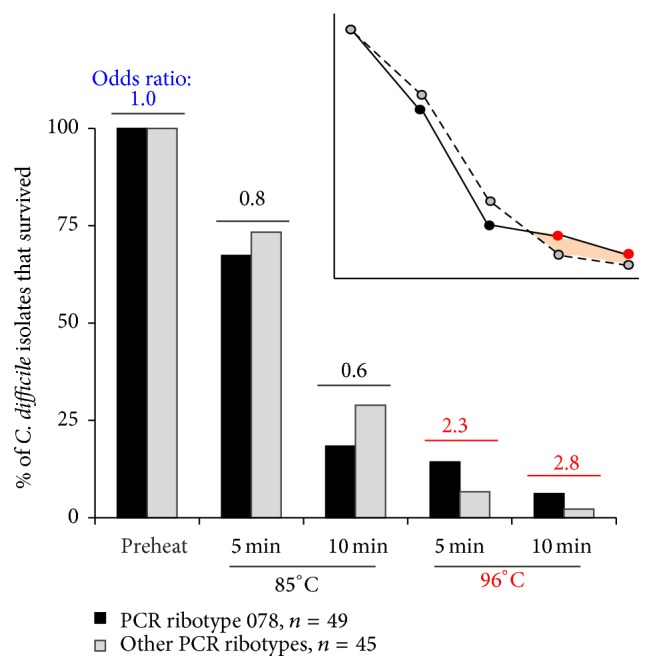
Thermal testing of a collection of* C. difficile* indicates temperature-dependent survival trends favoring PCR ribotype 078 strains at subboiling temperatures. Time and temperature of heating treatment of spore suspensions in PBS were significant predictors (*P* < 0.05) for spore survival reduction and switch in survival trends (odds ratio, OR). Comparing 078 with non-078 ribotypes, OR = 1 indicates that both groups would be equally likely to survive; OR > 1.0 indicated ribotype 078 was (2.3–2.8 times) more likely to survive (high heat, 96°C); OR < 1.0 indicated other non-078 PCR ribotypes would be would be (0.2–0.4 times; 1-OR) more likely to survive.

To determine if heat could favor the selection of other PCR ribotype 078 isolates, we analyzed the individual survival rates of 94 isolates from the mid-2000s (49 of ribotype 078 and 45 of other ribotypes; ratio, 1.08 : 1). Inhibition was significant as time and temperatures increased (logit *P* < 0.001), with 4.3% (4/97) of isolates surviving the hottest treatment (96°C for 10 minutes; Figure 1). Survival ratios for “ribotypes 078” : “other ribotypes” increased from 1 : 1 and 0.7 : 1 with heating at 85°C (for 5 and 10 minutes, resp.) to 2.3 : 1 and 3 : 1 with heating at 96°C (for 5 and 10 minutes, resp.) suggesting a nonlinear interaction. Multivariate regression analyses confirmed an interaction between time-temperature and strain type, making the models unstable to quantify the association between survival data and strain type. Univariate odds ratio estimations indicated the need of testing larger collection of isolates (≥4 times larger) to better characterize the interaction observed. Strain source was not associated with survival probability.

**Figure 2:**
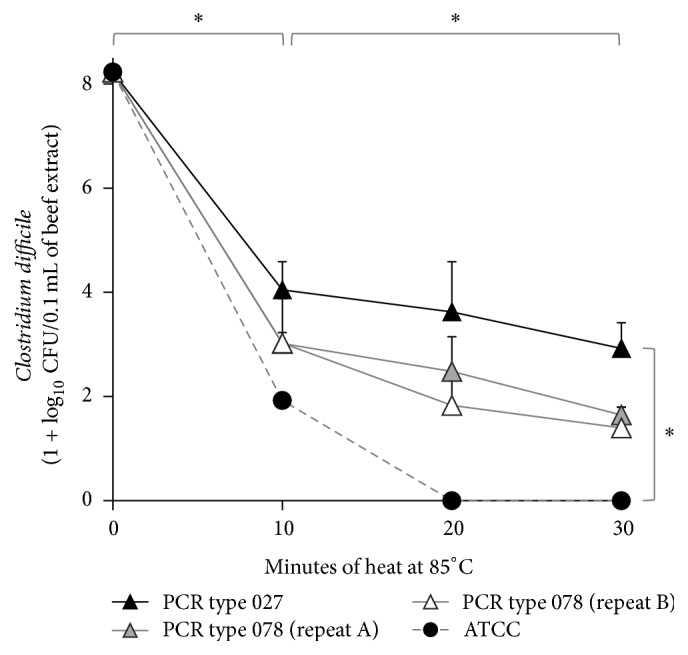
Thermal inhibition curves from* Clostridium difficile* mono-strain experiments in inoculated low-fat beef matrix. Thermal inhibition curves of prototypic* C. difficile* heated in 3% fat ground beef at 85°C. Asterisks, GLM *P* < 0.01. Upper bound error bars, standard deviation.

Next, individual heating of prototypic strains 027, 078 and ATCC-9689 in a lean beef matrix (instead of PBS alone) showed that 85°C significantly inhibited all strains. However, strain 027 was the most resistant and strain ATCC-9689 the most susceptible (4 and 6 log_10_ reduction within 10 minutes, resp.; GLM *P* < 0.001, Figure 2). At 85°C, further inhibition was observed only after 20 minutes of additional heat. Repeat testing with strain 078 showed optimal experiment reproducibility documenting intermediate heat susceptibility compared to strains 027 and ATCC-9689.

**Figure 3:**
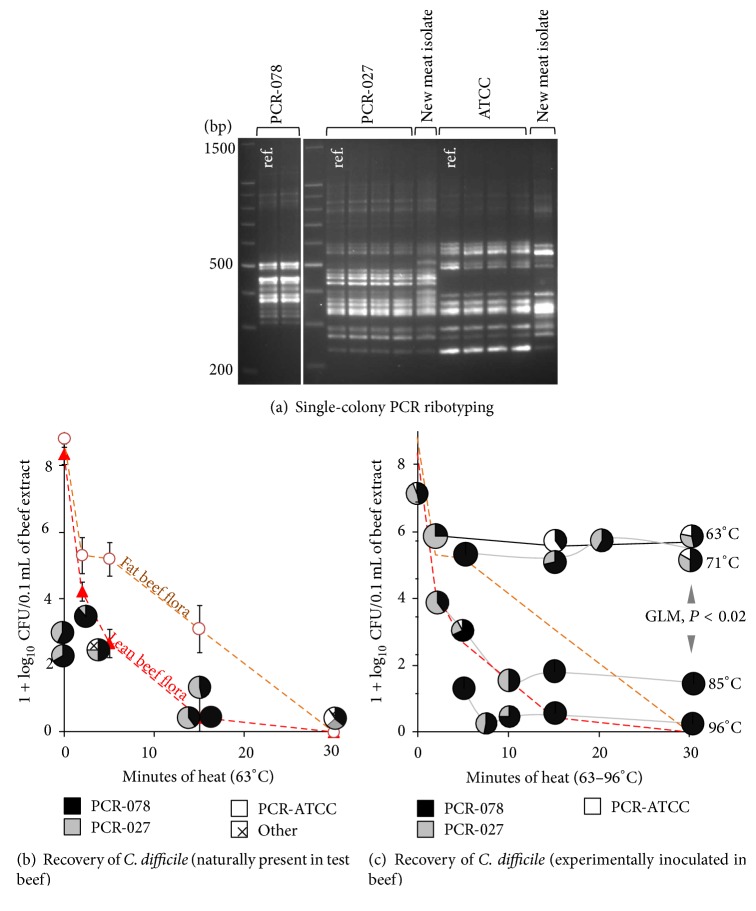
Moist heat resistance of* C. difficile,* naturally present and experimentally inoculated in retail ground beef, to various time-temperatures. Multistrain experiments in inoculated low 3% fat beef matrix. (a) Single-colony PCR ribotyping was used to subtype inoculated, naturally present, and heat-resistant experimentally isolates used (PCR 078, 027 and ATCC-9689). “PCR-”: prototypic strain. Note that other new meat isolates (PCR ribotypes) were naturally present in beef product purchased for the assay and are different from experimental isolates. (b) Single-colony PCR ribotyping based heat inhibition curves of* C. difficile* (pie charts connected with solid lines, average recovery frequencies) indicate presence of PCR 027 and 078 and other ribotypes naturally present in the meat package purchased for this assay (see panel a), at estimated concentrations of 2-3 log_10_ CFU/gram of contaminated raw ground beef tested. For comparison purposes, notice effect of pasteurization at 63°C on background indigenous anaerobic beef microbiota CFU isolated from two meat packages at different time points (dashed lines, mean ± SD, triplicate) for two fat concentrations 3 and 30%. (c) Single-colony PCR ribotyping heat inhibition curves of experimentally inoculated* C. difficile *indicate that PCR ribotype 078 would be more resistant to heat as temperature and time increase.

Lastly, in the experimental block design for the 1 : 1 : 1 strain mixture and natural contaminants, combined with single colony selection and PCR ribotyping of surviving spores (purified colony-forming units) show that both retail ground beef products (same manufacturer) used as heating matrices were naturally contaminated with up to 2.6 log_10_ · g^−1^ of* C. difficile*. This experiment also showed that our prototypic strains and two “new” ones (PCR ribotypes 027-like and ATCC-9689-like) were heterogeneously present in the ground beef based on direct culture of 1–3 g beef aliquots (1 : 1 PBS/meat, 100 *μ*L incubated in Tryptic Soy agar plate) (Figures 3(a)-3(b)). At 63°C, heat eliminated all other microbial indigenous beef flora with 7 log_10_ reduction within 30 minutes (dashed lines in Figure 3(b)), but not all naturally present* C. difficile*.

**Figure 4:**
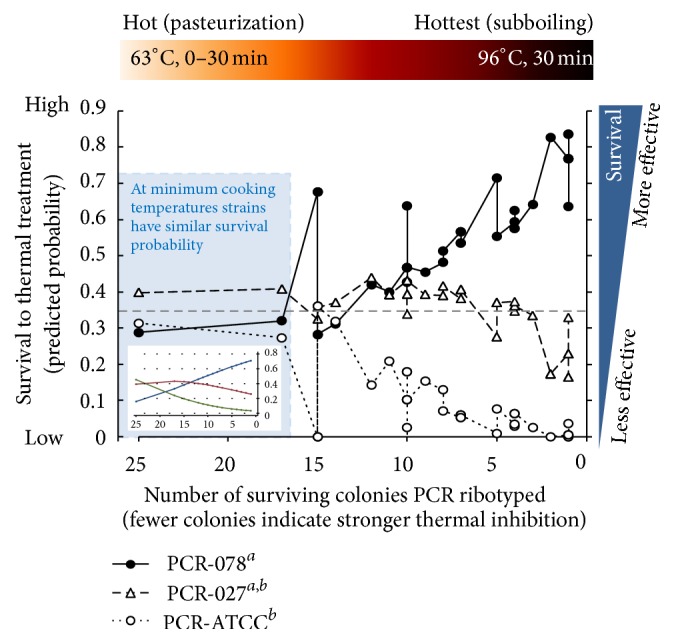
Predicted survival probabilities for three* C. difficile* genotypes show increased survival odds for PCR 078 as the number of survivor spores tested decreases as heat lethality increases. Raw data is from 1 : 1 : 1 strain mixture from Figure 3(c), plotted against the number of surviving colonies. The sum of probabilities at each value on the *x*-axis equals 1, up to 3 independent replicates for each *x*-axis value. Distinct superscripts indicate significant differences, multivariable multinomial logistic adjusted *P* < 0.01. Inset, plot of estimated smoothed probabilities of survival for the predicted data depicted in dot-line plot, without controlling for temperature.

Heat inhibition of* C. difficile* (1 : 1 : 1 mixture) in inoculated meat was temperature dependent. Compared to 63 and 71°C, which reduced only 1-2 log_10_ units within 30 minutes, 85 and 96°C were inhibitory within 10 minutes. Despite significant inhibition, up to 2 log_10_ of spores still remained viable after 30 minutes (GLM, *P* < 0.02, Figure 3(c)). Single-colony PCR ribotyping of survivor* C. difficile* indicated that heating at neither 63 nor 71°C affected the 1 : 1 : 1 population prevalence of the* C. difficile *mixture; all strains had equal probability of surviving 30 minutes of heat. However, at 85 and 96°C heat exerted a major selective effect, favoring PCR ribotype 078 while completely inhibiting ATCC-9689 spores (Figure 3(c), pie charts). Adjusted multivariable multinomial logistic regression quantitatively determined that the three strains had similar survival probabilities initially with sublethal heating, but outstanding strain selection occurred favoring PCR ribotype 078 as heat lethality increased (*P* < 0.01; Figure 4). There were no differences attributable to the fat content in beef.

## 4. Discussion

Here we hypothesized that thermal processing (cooking) destroys some* C. difficile* strains favoring the systematic selection of others. Our results support as proof-of-principle such heat selection theory among emerging strains of* C. difficile* concurrently heated in the same food item as thermal lethality increases. At 63°C, heat eliminated all non-*C. difficile* naturally occurring background microbiota with 7 log_10_ reduction within 30 minutes (as it is common with “batch” pasteurization) [32], indicating the absence of other heat-resistant or spore-forming microorganisms in the meat. Our study highlights the adequate food safety effect of 63°C for 30 min on the indigenous beef flora as a surrogate for vegetative pathogens [32], but it clearly shows that naturally present* C. difficile* can be inhibited but not eliminated from contaminated food. In this context, it is important to highlight that the meat packages used as beef matrix tested positive for* C. difficile* using direct culture of various beef aliquots. Thus, the prevalence of food contamination is expected to be high if product sampling intensifies, not only in meats [33], but also in vegetables [34] and hospital meals [35], as earlier suspected in a study using triplicate enrichment culture broths [36].

Our study also indicates the presence of a nonlinear interaction between time-temperatures and strain. At 85°C, strain PCR ribotype 027 could be selected over other strains including PCR ribotype 078 and ATCC-9689 strains tested. Unexpectedly temperatures reaching the water boiling point (~96°C) allowed PCR ribotype 078 to outsurvive the other tested strains, including PCR ribotype 027. Although PCR ribotyping might be a suboptimal predictor for heat resistance, study power analysis indicated that collections containing about 400 isolates would be needed in future experiments to test the external validity of the exact odds ratios estimated. Because our isolates had no recent history of heat treatments, our results indicate that genetics of the strains is critical predictor irrespective of phenotypic thermal memory in bacteria [24, 37].

Virtually most thermal studies designed to validate heating protocols to inhibit human pathogens are based on the overall effect of heat on a mixture (“cocktail”) of strains, the use of standard reference strains, or the use of nonpathogenic surrogates [32, 38, 39]. Because most enteric pathogens have fecal-oral transmission, foods are often involved as vehicles for transmission, and most of our foods are cooked, it is necessary to consider the potential that heat has as a selective force of increasingly virulent enteric pathogens, which may be present as dissimilar mixtures in retails foods, which upon heat processing could favor resistant and spore-formers. Studies comparing indigenous beef flora to reference vegetative foodborne pathogens indicate that pathogens are becoming more resistant to heat [32, 40] suggesting that such pathogens might have concurrently evolved heat tolerance by cooking-driven selection pressure.

We might be selecting emerging enteric pathogens without noticing. In the past, cooking with boiling water (90–100°C), as well as direct fire or pressure cookers (>100°C), was common. In modern times, there is increasing dependence on quick-to-cook foods, often prepared using minimal food safety cooking recommendations that did not affect the experimental probability of all* C. difficile *tested to survive in the food item used. Importantly, higher temperatures clearly favored in this study the selection of a prototypic PCR ribotype 078 isolated in 2004 [10, 14] compared to historic ATCC-9569 strain from the 1970s [29].

As we lower the cooking temperatures to attain desirable food texture and palatability, we may ingest more heat-injured spore-forming microorganisms, including* C. difficile* capable of recovering and regrowing [16, 41]. The continuous presence of foodborne infections associated with the consumption of cooked/undercooked foods [42–44], the remarkable association between heat-shock proteins and the potentiation of pathogenic traits in microbes [45], and the wide presence of* C. difficile* PCR ribotype 078 in the food supply [11, 19] indicate that pathogen selection during thermal processes could occur and be clinically relevant for species exposed to heated foods.

Because,* C. difficile* in foods is widely present at the retail level [36] and the infection dose in mouse models is very low and seemingly environmental [46, 47], it is important to elucidate the survival probability dynamics of* C. difficile* as a function of heat in contaminated items. It is possible that strain selection may occur in hospital settings during thermal disinfection of reusable materials (85°C for 1-2 minutes) [48], or in food production systems during thermal treatment of biosolids intended for land application [49].

Since the invention of fire, cooking facilitated hominins evolution by promoting socialization, maximizing food digestibility, and reducing foodborne infections [50–52]. However, it is uncertain how cooking may favor the selection of emerging pathogenic microorganisms. Because heat shock proteins increase antimicrobial resistance and virulence in pathogens [45], it is important to determine how heat selection modifies the epidemiology of increasingly problematic pathogens, including multidrug resistant hypervirulent* C. difficile* genotypes [33, 34, 53]. Alternatively, heat selection could help explain why some ribotypes from the 2000s, in particular PCR ribotype 027, have become less frequent in clinical cases with novel PCR ribotypes emerging over time in certain regions of Canada [54].

Compared to vegetative cells,* C. difficile* spores survive heating better than most potentially inhibitory background food microbiota; therefore further studies on the effect of heat processing on* C. difficile* intestinal colonization and virulence traits of survivor spores are warranted using animal models, including mice. Studying the probabilistic role of foodborne transmission in CDI directly in humans is obviously difficult; thus theoretical principles could further gain weight for future risk assessment purposes using hypothesis-testing* in vitro* and* in vivo* models.

## Figures and Tables

**Table 1:** Publicized minimum recommended cooking temperatures in North America to reduce exposure to foodborne pathogens.

Food category	Example items	Temperature	Rest time
Leftovers & casseroles	N/A	74°C (165°F)	None
Poultry	Chicken, turkey, duck, and goose; whole or parts	74°C (165°F)	None
Ground meats	Turkey, chicken	74°C (165°F)	None
	Beef, pork, veal, and lamb	71°C (160°F)	None

Fresh beef, veal, and lamb	Steaks, roasts, and chops	63°C (145°F)	3 minutes
Seafood	Fin fish, shrimp, lobster, and crabs, clams, oysters, and mussels; scallops	63°C (145°F) or cook until flesh is opaque and separates easily with a fork	None
Pork and ham	Fresh pork/raw ham	63°C (145°F)	3 minutes
	Precooked ham (to reheat)	60°C (140°F)	None

Adapted from U.S. cooking guidelines (http://www.foodsafety.gov/keep/charts/mintemp.html). In Canada, similar recommendations exist, except that whole and stuffed poultry should be cooked to at least 85°C (185°F) (see “safe internal cooking temperatures” at http://healthycanadians.gc.ca/). Rest time refers to the number of minutes needed at the recommended temperature to inhibit at least 6.5log_10_⁡ units of *Salmonella*, to be in compliance with the USDA performance standard for lethality (http://www.fsis.usda.gov/OPPDE/rdad/FRPubs/95-033F/95-033F_Appendix_A.htm) of the United States Department of Agriculture (USDA)—Food Safety and Inspection Service (USDA-FSIS) 9 CFR section 318.17(a)(1) (http://edocket.access.gpo.gov/cfr_2008/janqtr/9cfr318.17.html). Health Canada recently reduced the minimum internal cooking temperature recommendation for whole poultry from 85°C to 82°C based on *Salmonella* research [23].
